# Increasing oxygen deficiency changes rare and moderately abundant bacterial communities in coastal soft sediments

**DOI:** 10.1038/s41598-019-51432-1

**Published:** 2019-11-08

**Authors:** Hanna Sinkko, Iina Hepolehto, Christina Lyra, Johanna M. Rinta-Kanto, Anna Villnäs, Joanna Norkko, Alf Norkko, Sari Timonen

**Affiliations:** 1 Department of Microbiology, University of Helsinki, Viikinkaari 9, 00014 Helsinki, Finland; 2 Present Address: Department of Bacteriology and Immunology, University of Helsinki, Haartmaninkatu 3, Helsinki, 00014 Finland; 3 Institute of Environmental Medicine, Karolinska Institutet, Stockholm, Sweden; 4 Present Address: Faculty of Engineering and Natural Sciences, Tampere University, Tampere, Finland; 5 Tvärminne Zoological Station, University of Helsinki, Hanko, Finland; 6 Baltic Sea Centre, Stockholm University, Stockholm, Sweden

**Keywords:** Water microbiology, Microbial ecology

## Abstract

Coastal hypoxia is a major environmental problem worldwide. Hypoxia-induced changes in sediment bacterial communities harm marine ecosystems and alter biogeochemical cycles. Nevertheless, the resistance of sediment bacterial communities to hypoxic stress is unknown. We investigated changes in bacterial communities during hypoxic-anoxic disturbance by artificially inducing oxygen deficiency to the seafloor for 0, 3, 7, and 48 days, with subsequent molecular biological analyses. We further investigated relationships between bacterial communities, benthic macrofauna and nutrient effluxes across the sediment-water-interface during hypoxic-anoxic stress, considering differentially abundant operational taxonomic units (OTUs). The composition of the moderately abundant OTUs changed significantly after seven days of oxygen deficiency, while the abundant and rare OTUs first changed after 48 days. High bacterial diversity maintained the resistance of the communities during oxygen deficiency until it dropped after 48 days, likely due to anoxia-induced loss of macrofaunal diversity and bioturbation. Nutrient fluxes, especially ammonium, correlated positively with the moderate and rare OTUs, including potential sulfate reducers. Correlations may reflect bacteria-mediated nutrient effluxes that accelerate eutrophication. The study suggests that even slightly higher bottom-water oxygen concentrations, which could sustain macrofaunal bioturbation, enable bacterial communities to resist large compositional changes and decrease the harmful consequences of hypoxia in marine ecosystems.

## Introduction

Hypoxia, defined as <2 ppm or mg of dissolved oxygen l^−1 ^^1,2^, occurs naturally but infrequently on the coastal seafloors of semi-enclosed and stratified water bodies. Since the 1960s, levels of dissolved oxygen have drastically decreased in coastal areas due to anthropogenic loading of nutrients and organic matter and consequent eutrophication. More than 400 aquatic systems have been reported to suffer from eutrophication-driven severe anoxia and so-called “dead zones”^2–4^. Oxygen depletion has multiple deleterious effects on marine ecosystems and has been ranked as one of the major global environmental problems^3,5^. Oxygen depletion causes loss of invertebrate macrofauna, and disrupts the flow of food and energy from benthic microbial habitats to higher trophic levels^3,6^.

The composition of sediment bacterial communities clearly changes along oxygen or redox clines^7–11^. Hypoxia-induced changes in sediment microbial communities have direct and indirect consequences for biogeochemical cycles. For instance, in the absence of oxygen, nitrate, iron (Fe) or sulfate will be used as alternative electron acceptors by bacteria. The dissimilatory reduction of nitrate or Fe by microbes results in the release of ammonium and phosphate from sediment to the water column, where they can accelerate eutrophication^12,13^. In addition, microbial reduction of sulfate to hydrogen sulfide (H_2_S) increases the efflux of phosphorus as iron is bound to ferrous sulfide^12,14^.

Hypoxia-induced changes in sediment microbial communities may also harm the invertebrate benthic macrofauna in addition to the direct harmful effects oxygen deficiency has on macrobenthic communities^6,12,15^. In shallow, eutrophied coastal areas, the sediment sulfate-reduction zone is located close to the sediment-water interface^16^. There sulfate-reducing bacteria first cause habitat compression and eventually elimination of benthic fauna by producing toxic H_2_S^3^. The impaired benthic biodiversity and thus loss of bioturbating fauna further decreases transfer of oxygen into the sediment^17,18^. This, in turn, increases the release of redox-sensitive nutrients such as phosphorus from the sediment to the water column.

The loss of bioturbating fauna reduces the number of microbial habitats including oxic burrow walls, the faunal body surface, soft cell tissues and gut, thus decreasing microbial diversity in the sediments^19^. The diversity of microbial communities, i.e. richness and evenness, is generally thought to increase the stability of communities during disturbances^20^. Diverse communities are more buffered against disturbances than less diverse communities as they are likely comprised of taxa with complementary biological traits^20,21^. For example, Griffiths *et al*.^22^ observed that less diverse soil bacterial communities were functionally less resistant to long-term copper contamination.

Environmental microbial communities are generally uneven owing to the high number of rare species^20^, which have been hypothesized to serve as a seed bank in changing environmental conditions^23^. Thus, rare species may be essential in compositional turnover and adaptation along environmental gradients, such as increasing hypoxia. Although community resistance is a central concept in microbial ecology and is extensively studied, as reviewed recently^20^, the effect of differentially abundant fractions of microbial communities on overall community stability and ecosystem functioning remains unexplored. Only recently, due to advances in high-throughput sequencing methods, have studies determining the resistance of differentially abundant microbial fractions to environmental disturbances, such as in temperature and salinity, begun to appear^24,25^.

There is very little information about how sediment bacterial communities generally resist hypoxic stress *in situ* (however, see^26^). Moreover, to our knowledge, no study has investigated the stability of differentially abundant fractions of sediment bacterial communities during hypoxic-anoxic disturbance. It remains unknown how fast and to what extent sediment bacterial community composition changes during bottom-water oxygen deficiency. By performing a field experiment, we were able to acquire data for comprehensively testing for *in situ* changes in bacterial communities during short and long-term deoxygenation. We also considered differentially abundant fractions of the communities as they may differ in their respective capacity to resist large compositional changes and interact with surrounding ecosystem. Therefore, we further explored associations between differently abundant operational taxonomic units (OTUs) and the release of nutrients from the sediment to the water column (nutrient fluxes) as well as invertebrate benthic macrofauna. A comprehensive understanding of how oxygen deficiency impacts benthic microbial communities and their interactions with the surrounding ecosystem is essential for the assessment of coastal ecosystem sensitivity to eutrophication-driven hypoxia. It is also essential for the prediction of ecological consequences of short- and long-term hypoxic stress.

The resilience of bacterial community composition against oxygen deficiency were explored in subtidal sandy sediments of the Northern Baltic Sea. Experimental plots on the seafloor were artificially deoxygenated for shorter and longer periods (0, 3, 7 and 48 days). The variation in the structure and abundance of bacterial communities was determined with a combination of molecular methods, including high-throughput sequencing. To better understand the effect of oxygen deficiency on ecosystem functioning, we also analyzed the bacterial communities in relation to previously published data^17^ on nutrient fluxes and benthic fauna.

## Materials and Methods

### Research area and experimental setup

The field experiment was conducted on subtidal sandy sediments in the middle archipelago zone of the Hanko peninsula, northern Baltic Sea (59°50′44”N, 23°14′96”E). A detailed description of the research area and the experimental setup is provided by Villnäs *et al*.^17^. Briefly, soft-sediment habitats at 4 m depth were exposed to artificial oxygen deficiency by deoxygenating 1 m^2^ plots of the seafloor through securing plastic sheets onto the sediment for 3, 7, and 48 days during June and July, 2008. Such duration of hypoxic-anoxic disturbance is often caused by transient aggregations of drifting algal mats^27,28^ or temperature or salinity stratification^16^. Control plots (0-day deoxygenated) were not covered by plastic sheets. Four replicate plots of each treatment were positioned in a block design where replicates were separated from each other by a minimum of three meters. The deoxygenation was ended simultaneously for all treatments, as the plastics were gently rolled away in July, so that the *in situ* measurements and all sampling could be conducted under identical environmental conditions. Flushing of the sediment took place during 14 h after the plastics were rolled away to avoid initial sediment reactions. This period was not long enough for a complete re-oxidation, but it allowed the sediment to reach a quasi-stable state^29,30^. In the ambient permeable sandy sediments, representing the control, oxygen penetrated to several centimetres’ depth. Hypoxic and anoxic conditions with concurrent formation of H_2_S were reached after 1.5 and 7 days, respectively^17^.

### Nutrient fluxes, sediment sampling and benthic macrofauna

Nutrient fluxes were measured, sediment sampled and macrofauna identified as described in Villnäs *et al*.^17^. Briefly, the deoxygenation treatments were terminated by removing the plastic sheets, and samples were collected the following day to avoid sampling of only the initial sediment reactions. Nutrient fluxes were measured *in situ* using dark benthic chambers, and water samples were taken at start and end of the incubation that lasted 6.5 hours, ensuring that water oxygen levels within the chamber would remain ≥80%^17^. Thereafter, samples for sediment properties and benthic macrofauna were taken with cores (Ø 2.0 and 5.6 cm, respectively) from each replicate plot. After incubation, sediment samples were analysed for organic matter (OM), total C and N content as well as phosphate sorption properties. After sieving (0.5 mm), the benthic macrofauna was identified to species or genus level as described previously^17^. In addition, duplicate sediment samples were taken from the four replicate experimental plots per treatment by pushing a cut-off 30 ml plastic syringe into the sediment. After sample collection, the syringe was immediately sealed. The samples (n = 32) were stored at −20 °C until DNA extraction.

### DNA extraction

For DNA extraction, the first 3 cm of the surface sediment was aseptically cut from each frozen sediment sample, thawed and homogenized. If undisturbed, the upper 3 cm of sandy sediments at the sampling area are oxygenated, and were chosen for sampling to be able to compare the bacterial community compositions with previously published material^17^. Pore water was removed by centrifugation for 30 s at 10,000 *g* to allow accurate weighing of sediment. DNA was extracted from approximately 0.25 g of sediment using the PowerSoil^®^ DNA extraction Kit (MoBio Laboratories, Inc., Carlsbad, CA, USA) according to the manufacturer’s instructions. The extraction included a step which removes humic substances, which are known to inhibit PCR. DNA was eluted with DNA-free water.

### Molecular analyses

The DNA was used as a template to amplify bacterial 16S rRNA genes by PCR with FAM27f, labelled at the 59 terminus with 6-carboxyfluorescein (FAM- gagtttgatcmtggctcag)^31^ and 1405r (acgggcggtgtgta) (Oligomer Oy), modified from 1406r^32^ as described previously^8^. However, with the exceptions that the MgCl_2_ concentration was optimized to 2 mM, the amount of DNA was 15–25 ng and the total volume of a PCR reaction was 50 µl. PCR amplicons (100 ng), purified using QIAquick^®^ PCR purification Kit (Qiagen, Hilden, Germany), were used for terminal restriction fragment length polymorphism (T-RFLP) analysis^33^. T-RFLP was performed as described by Sinkko *et al*.^8^, with the exception that only the restriction endonucleases HaeIII and RsaI (Promega Corporation, Madison, WI, USA) were used to digest the 16S rRNA gene. The normalization procedure of terminal restriction fragments (T-RF)^34^ resulted in separate matrices of T-RFs created by HaeIII and RsaI with a length range of 27–700 bp. Four-fold standard deviation was used as a threshold for distinguishing background ‘noise’ from a ‘true’ signal. The numbers of bacterial 16S rRNA gene copies in the sediments were quantified from DNA by quantitative real-time PCR (qPCR) with primers Eub338 and Eub518^35^ as previously described^36^.

### Sequencing 16S rRNA gene

The V1–V3 region of the bacterial 16S rRNA gene was sequenced from a subset of DNA samples (n = 20, Supplementary Fig. 3) in a partial (22.4%; 6 566 126 raw reads) Illumina® MiSeq run in the Institute of Biotechnology, University of Helsinki. Prior to sequencing, two-step PCR was used to amplify the V1–V3 region, using the primers F27^37^ and pD′^38^, which contained at their 5′ ends the partial TruSeq adapter sequences ATCTACACTCTTTCCCTACACGACGCTCTTCCGATCT and GTGACTGGAGTTCAGACGTGTGCTCTTCCGATCT, respectively. The first PCR product was used as a template in the second PCR with full-length TruSeq P5 and Index containing P7 adapters.

### Analysis of 16S rRNA gene sequences

After sequencing, general read quality was assessed by the FastQC software (http://www.bioinformatics.babraham.ac.uk/projects/fastqc/) and the adapter and barcode sequences were removed from raw 16S rRNA gene sequences using Cutadapt^39^. The sequences were trimmed, denoised, aligned, taxonomically assigned and clustered to OTUs with mothur v1.34.1^40^ according to the standard operating procedure (SOP, http://www.mothur.org/wiki/MiSeq_SOP, accessed January 2015)^41^ (Kozich *et al*., 2013). Default settings were used with one exception: To increase the quality of trimming, the screen.seqs command was first executed to remove contigs in which the length of the overlapping region of forward and reverse reads was less than 25 bp. The second run of the screen.seqs command, removal of ambiguous and homopolymeric sequences, and further commands were used according to the SOP. Chimeric sequences were removed using UCHIME^42^ as implemented by mothur. Denoised 16S rRNA gene sequences were aligned against the Silva reference alignment (release 119, http://www.mothur.org/wiki/Silva_reference_files)^43,44^. 16S rRNA gene primers for the V1–V3 regions were removed from the aligned sequences using the options “start” and “end” of the screen.seqs command and the subsequent filter.seqs command. Aligned and trimmed 16S rRNA gene sequences were taxonomically assigned using reference 16S rRNA gene sequences from the Ribosomal Database Project (Release 11.3, available at http://www.mothur.org/wiki/RDP_reference_files)^45^, with a bootstrap confidence threshold of 80%. OTUs were clustered using a threshold of 97% similarity between sequences.

Sequence counts, included in OTUs, were normalized by variance stabilizing transformation (VST) in the R environment (R Development Core Team, 2015), using the function varianceStabilizingTransformation with default settings in the DEseq2 package^46^. This function removed an effect of varying sequencing depth on sample-wise sequence counts using size factors calculated from the observed library sizes. It also transformed sample-to-sample variation, which varied more than expected relative to Gamma-Poisson (negative binomial) distribution, by correcting variance estimates of each OTU within treatment on the log_2_ scale. After removing chloroplasts (15.1%), mainly of the diatoms *Bacillariophyta*, the final data was comprised of 1 818 946 high- quality bacterial 16S rRNA gene sequences that were clustered into 19 777 OTUs. Normalized counts were used in the statistical analyses.

### Nucleotide sequence accession numbers

Raw sequence reads have been deposited in the NCBI Sequence Read Archive (SRA) under the accession number SRP083830.

### Categories of abundant, moderate and rare OTUs

**Figure 1:**
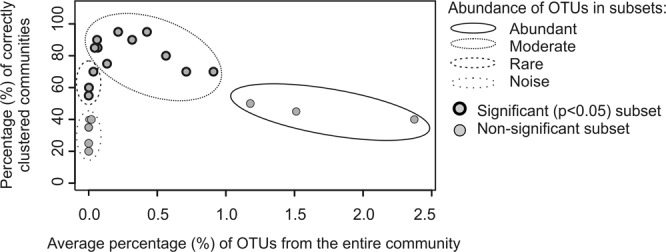
Nonlinear response of differently abundant bacterial communities to deoxygenation. The entire 16S rRNA gene sequence data was divided to 22 subsets of decreasing OTU abundance (Refer the chapter Categories of abundant, moderate and rare OTUs). Each subset thus contained a part of the total OTUs of all bacterial communities (samples). The percentage of correctly clustered communities (y-axis) describes how well bacterial communities in each subset clustered based on the duration of deoxygenation (0, 3, 7, and 48 days) in the discriminant analysis, based on Bray- Curtis dissimilarity. The higher the percentage of correctly clustered communities, the better the bacterial communities clustered based on the duration of deoxygenation, i.e. the more sensitive they were to hypoxia.

Most OTUs were present in all treatments but their abundance varied. To examine how differentially abundant fractions of bacterial communities responded to oxygen deficiency, the total normalized 16S rRNA gene sequence data was divided randomly to subsets (n = 22) of gradually decreasing OTU abundance (Fig. 1). The mean abundance of an individual OTU in the subsets ranged from 2.4 to 0.00005%. Each subset was examined by distance-based and nonparametric discriminant analyses (db-DA, more details in statistical analyses)^47,48^ to determine how well differentially abundant fractions were clustered based on the duration of the deoxygenation (i.e. how strongly they responded to oxygen deficiency). As the response of OTUs depended on their abundance, we combined subsets of OTUs with fairly equal mean abundance and response to deoxygenation to produce abundant, moderate, rare and very rare (i.e. noisy) fractions of the communities (Fig. 1). These fractions, excluding the noisy OTUs, were used in further analyses. Recent investigations have principally explored the abundant or rare microbial fractions of the microbial communities (reviewed e.g. by^23,49^) but often disregarded the moderate fraction. By analyzing these three fractions we could distinguish hypoxia- and anoxia-mediated compositional changes in bacterial communities.

In the abundant fraction, each OTU covered more than 0.5% of the entire community, corresponding to a minimum of 8 649 sequences (Supplementary Fig. 1). Each moderate OTU accounted for less than 0.5% (<8 649 sequences) but more than or equal to 0.001% (≥22 sequences) of the entire community. Each rare OTUs accounted for less than 0.001% of the entire community. The rarest OTUs, such as singletons, covered less than 0.0001% of the total community or possessed a significant amount of uncertainty in the proportion of counting reads (Poisson noise) (Supplementary Fig. 1).

### Diversity of T-RFs, 16S rRNA gene sequences and macrofauna

The Shannon H’ diversity index, i.e. Shannon H with natural logarithm, was calculated for T-RFs, 16S rRNA gene sequences and macrofauna in the R package vegan using the function diversity^50^. As T-RFs originating from chloroplasts or bacterial 16S rRNA genes could not be exclusively separated from each other, 16S rRNA gene sequences assigned to chloroplasts (n = 302 998) were included in the Shannon index together with bacterial 16S rRNA gene sequences (n = 2 113 037). This allowed us to compare the diversity of T-RFLP and 16S rRNA gene sequence data. OTUs including only one sequence were excluded before calculations.

### Statistical analyses

All statistical analyses were performed in the R environment. Differences in 16S rRNA gene copy number between treatments were tested by the nonparametric (rank-based) Kruskal-Wallis test in the R package stats. Differences in bacterial community composition between treatments were tested based on T-RFs and 16S rRNA gene sequences by distance-based and permutational multivariate analysis of variance (permutational MANOVA) using the function adonis in the package vegan^50,51^. Discrimination between the bacterial community compositions and their *a priori* classification according to the duration of deoxygenation was tested by the db-DA^47,48^ using the function CAPdiscrim in the package BiodiversityR^52^. The function add.spec.scores was used to calculate the discriminant axes scores of OTUs, which were used to visualize OTUs onto the db-DA ordinations, produced by the first and second linear discriminant axes scores. Homogeneity of group variances, assumed by both the permutational MANOVA and the db-DA, were tested using the function betadisper in the package vegan. In all distance-based analyses, sample-wise distances were calculated using Bray-Curtis dissimilarity and 9999 permutations were used to calculate significance. In addition, we tested whether each OTU varied significantly during the deoxygenation treatments using the Wald test in the package DESeq2^46^.

To study relationships between 16S rRNA gene sequences and nutrient fluxes as well as sediment oxygen consumption, Pearson correlations between OTUs and ammonium, silicate, iron and oxygen fluxes were calculated using the function cor in the package stats. The nutrient fluxes had been previously determined by Villnäs *et al*.^17^. Only those OTUs which were associated with nutrient fluxes (with a strict alpha level of *p* < 0.001) in the marginal tests for a distance-based linear model, formulated by the adonis function in the package vegan, were included in the correlation analysis.

The relationship between the diversity of the 16S rRNA genes and macrofauna, calculated by Shannon H’, were investigated by general linear models using the function lm in the package stats. The *p*-values were calculated using permutations of the function adonis in the package vegan, based on Euclidean distances between samples.

## Results

### Effects of *in situ* deoxygenation on bacterial communities in Baltic sandy sediments

#### Quantity of bacterial 16S rRNA genes

The quantity of bacterial 16S rRNA genes varied between 5.24 × 10^6^ copies g^−1^ and 2.38 × 10^8^ copies g^−1^ in sediments exposed to deoxygenation. The 16S rRNA gene copies in control (0-day treatment) sediments had a mean of 6.16 × 10^7^ copies g^−1^. After three days of deoxygenation, the mean number of 16S rRNA gene copies decreased to 2.09 × 10^7^ copies g^−1^, but increased slightly after seven days of deoxygenation (2.81 × 10^7^ copies g^−1^). After 48 days of deoxygenation, the number of 16S rRNA genes further increased (3.85 × 10^7^ copies g^−1^). Nevertheless, the number of 16S rRNA gene copies did not differ significantly (*p* = 0.122) between treatments (Supplementary Fig. 2).

#### Variation in terminal restriction fragments of 16S rRNA genes

The restriction endonucleases HaeIII and RsaI created 88 and 64 T-RFs above the thresholds, respectively. The deoxygenation treatments explained 67% and 71% of the variation in T-RFs created by HaeIII and RsaI, respectively. The T-RFs created by both HaeIII and RsaI significantly changed after 7 and 48 days of deoxygenation (Supplementary Table 1). Specifically, 78% (*p* = 0.01) of the T-RF compositions created by HaeIII and 84% (*p* = 0.01) of the T-RF compositions created by RsaI were correctly discriminated according to the deoxygenation treatments (Supplementary Fig. 3). The treatment-wise differences in T-RF compositions created by HaeIII may have been blurred due to their increased variances (Supplementary Fig. 4).

#### Stability of differently abundant communities of 16S rRNA gene sequences during deoxygenation

70% of the communities of the 16S rRNA gene sequence data clustered according to the duration of deoxygenation (Supplementary Fig. 5B). However, by analysing subsets of OTUs with decreasing OTU abundance, we found that differentially abundant OTUs responded dissimilarly to deoxygenation (Fig. 1). The subsets of abundant OTUs did not significantly differ based on the duration of deoxygenation, while the subsets of moderate OTUs (75% to 95%) clustered well (Fig. 1). The subsets of rare OTUs also differed significantly between treatments, but the percentage of subsets that clustered based on the duration of deoxygenation was lower than for moderate communities (55–75%).

These results indicate that abundant OTUs resisted oxygen deficiency, while rare, and in particular, moderate OTUs were sensitive. Therefore, subsets of the abundant, moderate and rare OTUs were combined (Fig. 1) to form the abundant, moderate and rare fractions of communities. The fractions were further examined, along with of the total community, to better distinguish changes in bacterial communities mediated by oxygen deficiency.

#### Composition and changes in abundant, moderate and rare communities mediated by oxygen deficiency

Among the abundant OTUs, the bacterial 16S rRNA gene composition did not differ between treatments after the correction of *p*-values (Table 1). Of all abundant bacterial communities, only those which were deoxygenated for 48 days clustered loosely together (Supplementary Fig. 5A). Consequently, different OTUs were fairly evenly distributed during deoxygenation. Taken together, the abundant OTUs covered 43% of the entire community and represented mainly the actinobacterial, deltaproteobacterial and gammaproteobacterial classes (Supplementary Fig. 6).

**Table 1:** Global analysis of variance and pairwise comparisons of bacterial communities between different durations of deoxygenation (treatments).

Treatments	Sums of squares	Mean squares	F Model	R2	Pr (>F)	P adjusted
**Abundant OTUs**						
0 d vs. 3 d	0.0000	0.0000	0.37	0.06	0.89	0.89
0 d vs. 7 d	0.0001	0.0001	0.59	0.09	0.77	0.89
0 d vs. 48 d	0.0002	0.0002	3.32	0.36	**0.03**	0.09
3 d vs. 7 d	0.0001	0.0001	0.56	0.09	0.80	0.89
3 d vs. 48 d	0.0002	0.0002	2.71	0.31	0.06	0.12
7 d vs. 48 d	0.0002	0.0002	3.07	0.34	**0.03**	0.09
Global test	0.0004	0.0001	1.55	0.28	0.11	
**Moderate OTUs**						
0 d vs. 3 d	0.0002	0.0002	0.90	0.13	0.807	0.80
0 d vs. 7 d	0.0002	0.0002	1.14	0.16	0.144	0.21
0 d vs. 48 d	0.0006	0.0006	2.92	0.33	**0.029**	**0.05**
3 d vs. 7 d	0.0002	0.0002	0.98	0.14	0.570	0.69
3 d vs. 48 d	0.0005	0.0005	2.20	0.27	**0.029**	**0.05**
7 d vs. 48 d	0.0005	0.0005	2.56	0.30	**0.025**	**0.05**
Global test	0.0011	0.0004	1.77	0.31	**0.001**	
**Rare OTUs**						
0 d vs. 3 d	0.0001	0.0001	0.97	0.14	0.659	0.79
0 d vs. 7 d	0.0001	0.0001	0.94	0.14	0.892	0.88
0 d vs. 48 d	0.0001	0.0001	1.49	0.20	**0.026**	0.06*
3 d vs. 7 d	0.0001	0.0001	1.00	0.14	0.430	0.65
3 d vs. 48 d	0.0001	0.0001	1.32	0.18	**0.029**	0.06*
7 d vs. 48 d	0.0001	0.0001	1.40	0.19	**0.028**	0.06*
Global test	0.0003	0.0001	1.20	0.23	**0.004**	

Bacterial community composition was based on the abundant, moderate and rare OTUs of 16S rRNA gene sequences. Mean abundances of each OTUs were calculated from within - treatment plot duplicates before the analysis. The variance analysis used Bray-Curtis dissimilarity between samples and 9999 permutations to calculate *p*-values. *P* values were adjusted using the false discovery rate (FDR) method. Significant *p*-values are bolded and marginally significant marked with a star.

**Figure 2:**
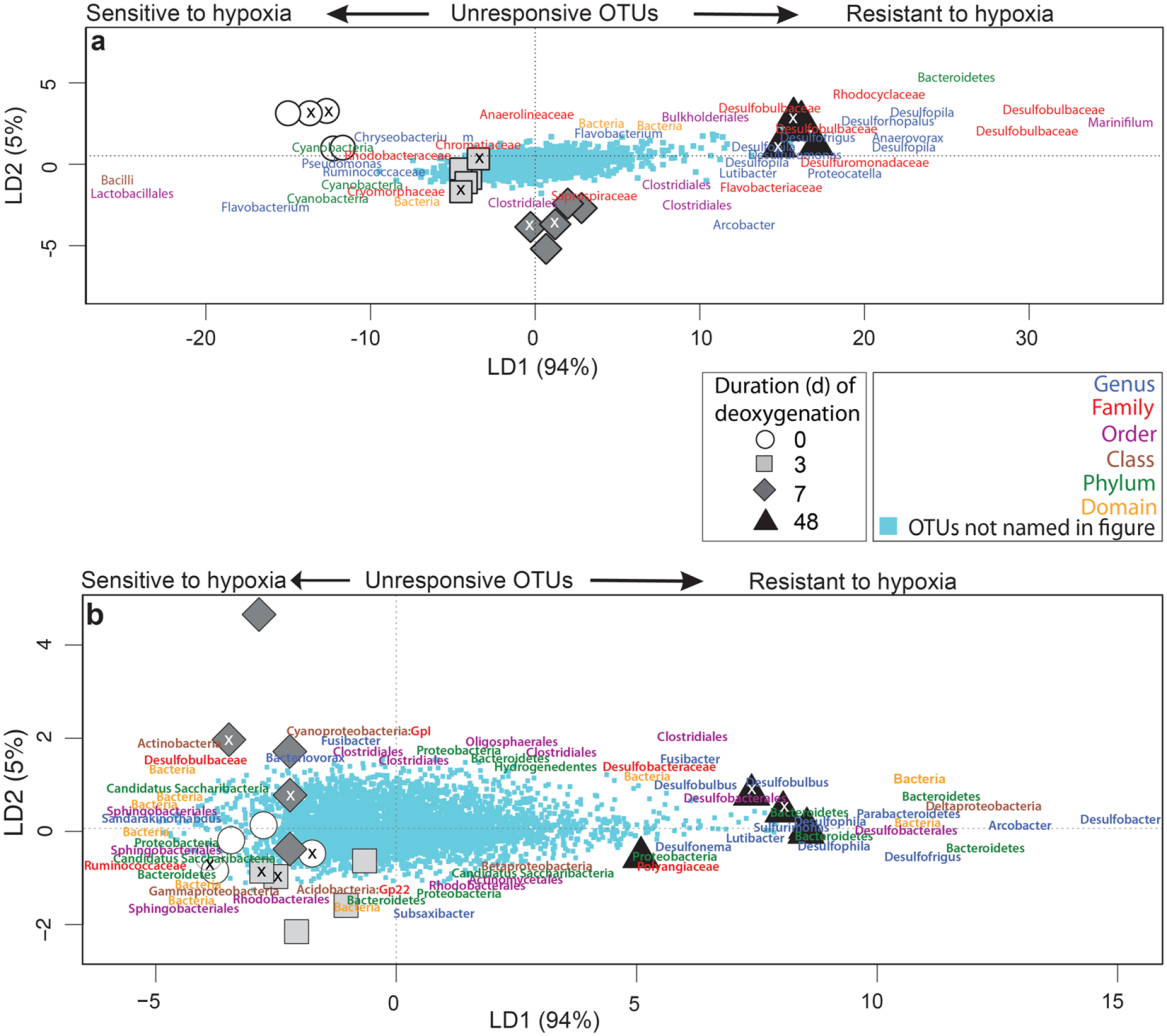
Differences between bacterial communities during deoxygenation of the seafloor. Ordination plots for (**a**) moderate and (**b**) rare OTUs of bacterial 16S rRNA genes were constructed using scores of linear discriminant axes (LD) 1 and 2 produced by nonparametric and Bray-Curtis dissimilarity based discriminant analysis. Only the best discriminating bacterial OTUs are named. (**a**) 100% of the community compositions (n = 20) were correctly classified (*p* = 0.0001) based on the duration of deoxygenation. The discriminant analysis used 12 principal coordinates capturing the largest amount of variation (71% of total variation). (**b**) 75% of the community compositions (n = 20) were correctly classified based on the duration of deoxygenation (*p* = 0.0001). 7 principal coordinates, which explained 46% of the total variation, were used in the discriminant analysis. Ticks on the top of symbols mark within – treatment plot replicates showing their variation.

Among the moderate OTUs, the 16S rRNA gene composition of the 0-, 3- and 7-day treatments differed from 48-day treatment (Table 1). Bacterial communities formed distinct clusters based on the duration of deoxygenation (Fig. 2a). OTUs assigned to potentially sulfate reducing taxa such as the family Desulfobulbaceae and its genus *Desulfophila*, and OTUs of the order Clostridiales, e.g. the genera *Anaerovorax* and *Proteocatella* particularly increased during prolonged deoxygenation (Fig. 2a). Some moderate OTUs such as *Pseudomonas* seemed to be sensitive to deoxygenation and occurred more frequently in control sediments (Fig. 2a). Taken together, the moderate OTUs covered 53% of the entire community and represented mainly the same taxonomic classes as the abundant OTUs but had a larger proportion of Planctomycetia, Anaerolineae and Alphaproteobacteria (Supplementary Fig. 6).

The compositions of the rare OTUs clustered fairly well based on deoxygenation (Fig. 2b), and the 48-day treatment differed marginally from the 0-, 3- and 7- day treatment (Table 1). Generally, the rare OTUs explained less of the variation in the communities than the moderate OTUs. Among rare OTUs, taxa assigned to the phylum Bacteroidetes such as the genus *Parabacteroidetes* as well as potential sulfate reducing taxa such as the genera *Desulfofrigus*, *Desulfophila*, and *Desulfobacter* increased during deoxygenation. (Fig. 2b). The diversity of Deltaproteobacteria was notably high among rare OTUs (Supplementary Fig. 6).

The majority of the OTUs in the abundant, moderate and rare fractions were located close to the origin of the ordination plots (Supplementary Fig. 5A, Fig. 2a,b), i.e. they were fairly equally abundant in every treatment. Based on the Wald test, only 2% (215 OTUs) varied significantly (*p* < 0.05) across the treatments, and only a minority (4.7%, 10 OTUs) of these OTUs belonged to the abundant fraction of the community.

#### Relationships between bacterial communities and nutrient effluxes across the sediment-water-interface

Differences in nutrient effluxes were primarily found for ammonium (NH_4_^+^), silicate (Si) and iron (Fe^2+^), which all showed an increasing efflux with the duration of deoxygenation^17^. The ammonium efflux was on average 8.1 ± 2.9 (SE) µmol m^−2^ h^−1^ in control sediments, while effluxes in the 7- and 48-day treatment were 9 and 12 times higher, respectively, and thus deviated significantly from the control treatment (one-way ANOVA; p < 0.001). Likewise, the silica efflux was lowest in the control treatment (6.1 ± 3.0 µmol m^−2^ h^−1^), and while a fourfold increase was observed after the 3- and 7- day treatment, a ten-fold increase compared to the control was measured after 48 days of deoxygenation (one-way ANOVA; p < 0.001). No significant differences were found between treatments for the iron efflux, but values after 7 and 48 days of deoxygenation were 1.8 and 2.7 times higher compared to the control. Low effluxes were observed for both NO_3_^−^ + NO_2_^−^ and PO_4_^3−^ (<4 µmol m^−2^ h^−1^) in all treatments. Oxygen was consumed in all treatments, and the highest influx was noted for undisturbed sediments (−33.3 ± 1.5 mg m^−2^ h^−1^). The oxygen consumption decreased in the 3- and 7- day treatments with, on average, 4 and 10 mg m^−2^ h^−1^ compared to the control, while a slightly increasing consumption was observed after 48 days of deoxygenation (cf. Villnäs *et al*.^17^).

**Figure 3:**
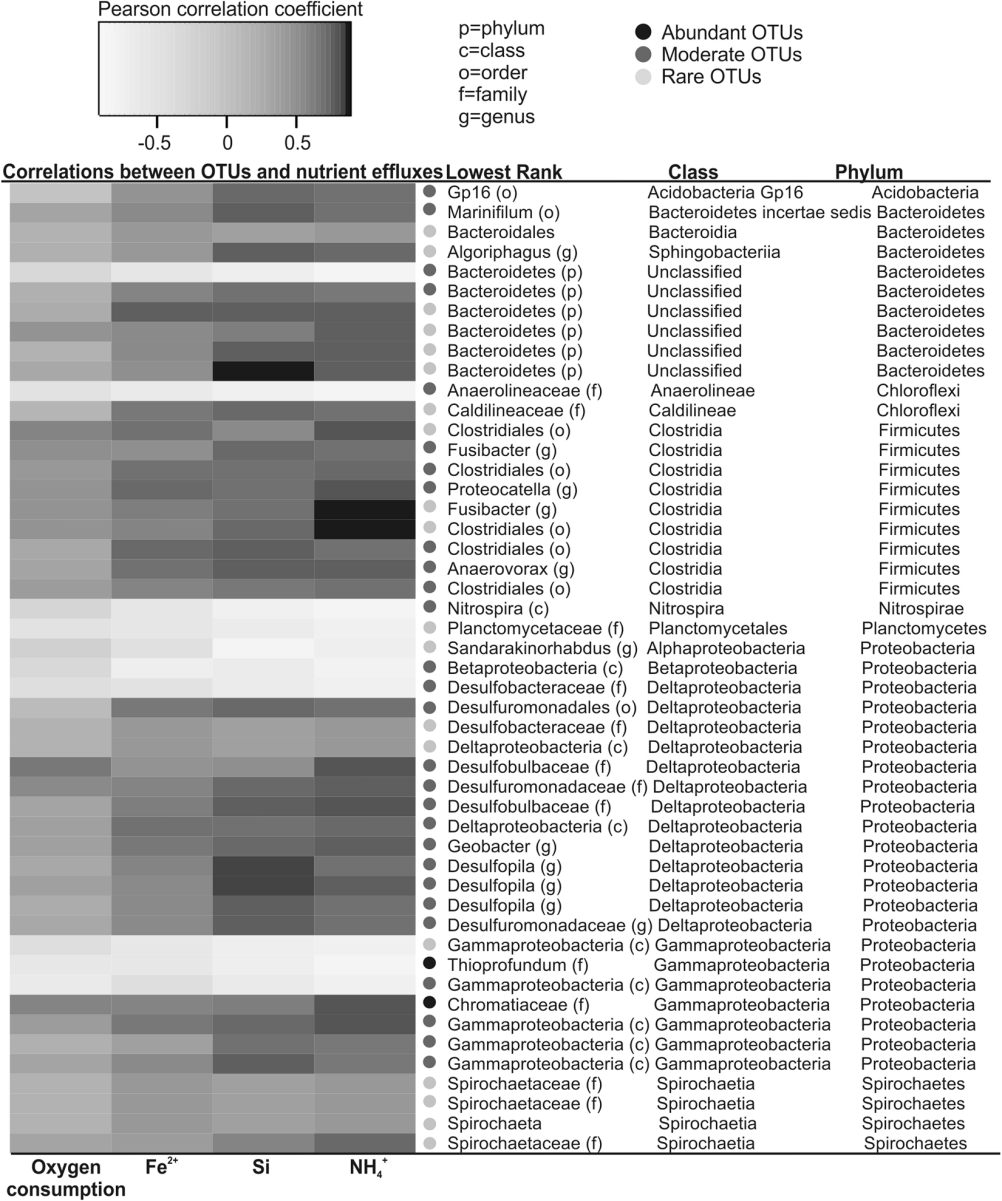
Relationships between bacterial community composition and nutrient effluxes across the sediment-water-interface. Pearson correlations were calculated between operational taxonomic units (OTUs) of 16S rRNA genes and oxygen consumption as well as fluxes of soluble iron (Fe^2+^), silicate (Si) and ammonium (NH_4_^+^). The darker the color, the higher the correlation (see the scale from the upper part of the figure). Only those OTUs are shown which were most significantly (*p* < 0.001) correlated with nutrient fluxes in regression analyses and were identified at least on the phylum level.

There were 58 OTUs that were significantly (α: *p* < 0.001) correlated with nutrient effluxes across the sediment-water interface. The majority (75.9%) of these OTUs correlated positively with iron, silicate and particularly ammonium fluxes (Fig. 3). Most of the positively correlated OTUs belonged to the rare (39.6%) and moderately abundant communities (32.8%). Only 1.7% of the OTUs correlating with nutrient fluxes were abundant. OTUs belonging to the class *Clostridia* such as the genus *Fusibacter*, the class *Deltaproteobacteria* such as the family *Desulfobulbaceae*, and the class *Bacteroidetes* had the highest positive correlation with ammonium (Fig. 3). OTUs of the phylum *Bacteroidetes* and the genus *Desulfophila* correlated positively with the silicate flux. The iron flux correlated positively mainly with taxa in the classes *Clostridia* and *Deltaproteobacteria*. Oxygen consumption correlated positively mainly with the classes *Clostridia* and certain members of *Deltaproteobacteria* such as the family *Desulfobulbaceae* (Fig. 3).

#### Diversity of bacterial communities and benthic macrofauna affected by oxygen deficiency

**Figure 4:**
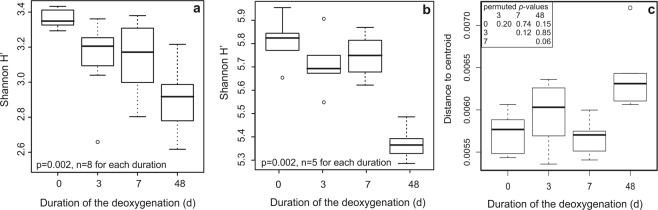
Variation in alpha and beta diversity during deoxygenation of the seafloor. Variation in Shannon H’ indexes for (**a**) terminal restriction fragments created by HaeIII (n = 88) and (**b**) 16S rRNA gene sequences (n = 2 113 037) as well as **(c)** treatment-wise Bray-Curtis distances of 16S rRNA gene sequences. The solid line depicts the median. The mean of Shannon H’ is positioned in the middle of the box.

The Shannon H’ index of T-RFs created by HaeIII (Fig. 4a) and the total bacterial 16S rRNA gene sequences (Fig. 4b) decreased significantly during the deoxygenation. The diversity of the total 16S rRNA gene sequence data formed a sigmoidal pattern, similar to those of the abundant and rare fractions but different than the pattern of the moderate fraction (Supplementary Fig. 7). Contrary to the alpha diversity (Shannon H’) of the total bacterial communities, beta diversity increased in the 3- and 48-day treatment (Fig. 4c).

**Figure 5:**
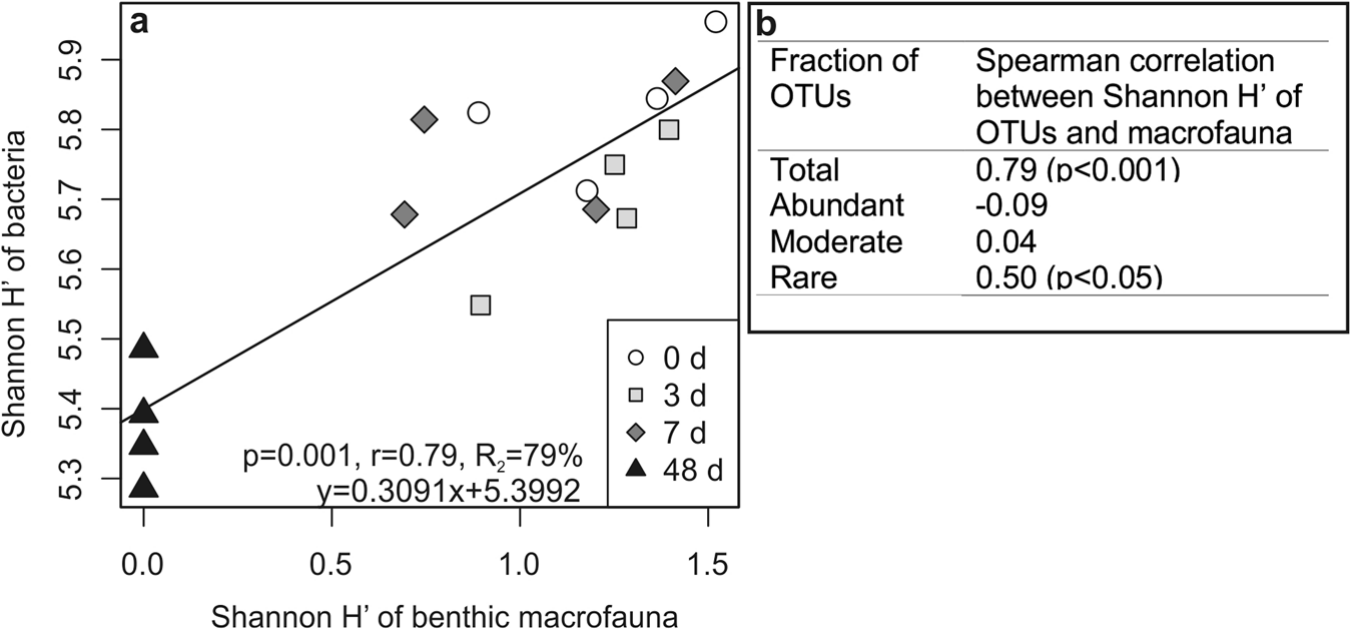
Relationship between diversity of bacteria and benthic macrofauna during deoxygenation. (**a**) Linear regression of Shannon H’ of total bacterial community compositions and benthic macrofauna. (**b**) Spearman rank correlation between Shannon H’ of differently abundant community compositions and benthic macrofauna.

The number of macrobenthic species was low in the control sediments (≤6 species), and exhibited a gradual, non-linear decline in response to increased hypoxic-anoxic disturbance (cf. Villnäs *et al*.^17^). Abundances measured >10 000 ind. m^−2^ in the 0 and 3-day treatments but significantly lower numbers were observed in the 7-day treatment (on average 1518 ± 284 ind. m^−2^) while no living macrofauna was observed after 48 days of deoxygenation. A more gradual decline was observed for benthic biomass with increased duration of deoxygenation, due to the occasional occurrence of surviving large bivalves, such as *Macoma balthica* and *Mya arenaria*. Bacterial alpha diversity was correlated (*p* = 0.001) with the diversity (H’) of benthic animals that explained 77% of the variation in bacterial diversity (Fig. 5a). However, 73% of the effect of faunal diversity overlapped with the effect of the duration of deoxygenation (Supplementary Fig. 8). The direct effect of faunal diversity on bacterial diversity was 3%. Rare OTUs contributed significantly to the correlation between faunal and bacterial diversity (Fig. 5b).

## Discussion

For the first time, we show experimentally *in situ* that oxygen deficiency has a distinct impact on differentially abundant fractions of sediment bacterial communities. The abundant bacterial fraction persisted unchanged under deoxygenation for a considerably longer time than the moderate fraction. Importantly, we show that the compositional changes are related to ecosystem functioning. Specifically, moderate and rare OTUs are associated with increasing nutrient effluxes and rare OTUs with the diversity of benthic macrofauna. Our study provides new information on how oxygen deficiency affects benthic microbial communities and their interactions with the surrounding ecosystem. These results help to estimate the capacity of sediment bacterial communities to tolerate hypoxia and the damage large compositional changes can inflict on the ecosystem. This information is essential for the assessment of coastal ecosystem sensitivity to eutrophication-driven oxygen deficiency and the ecological consequences of short- and long-term hypoxic stress.

### Oxygen deficiency altered bacterial community composition but not the number of bacteria

Oxygen depletion, resulting in low redox potential in sediments, caused significant changes in bacterial community composition, although it did not affect the abundance of bacterial 16S rRNA gene copies. To our knowledge, only one previous experiment has examined the *in situ* response of bacterial community composition to hypoxic stress in sediment^26^. In the study, carried out by denaturing gradient gel electrophoresis, the community compositions of deoxygenated (defaunated) and ambient sediments did not change after 25 days of deoxygenation. In our study, the OTU resolution of T-RFLP and high-throughput sequencing were substantially higher, allowing us to detect significant changes in bacterial communities already after seven days of oxygen deficiency.

Higher resolution is needed, as the majority (98%) of OTUs did not significantly react to presence or absence of oxygen. The high proportion of unresponsive OTUs is expected, as the majority of bacteria in coastal sandy sediments are either dead (ca. 70%) or dormant (ca. 28–29%), and only about 1.5% of bacteria are active^53^. Here, 2% of all OTUs varied significantly across treatments. Therefore, only a small portion of all OTUs in our study may represent cells which actively adapted to the decreasing oxygen conditions and subsequent low redox conditions. Also, within oxic sediment layers there are anoxic microenvironments with anaerobic bacteria, which do not react to absence of oxygen, and can partly explain a small portion of reactive OTUs.

Consistent with our observations, the composition of bacterial communities clearly changed along a natural spatial gradient of decreasing redox potential in Baltic Sea and Black Sea sediments^7,11^. Other studies, using Baltic Sea sediment *in vitro*, showed a significant response in bacterial community composition to manipulated redox potential^10,54^. However, Steenbergh *et al*.^10^ suspected that uncontrolled environmental factors caused overlapping of bacterial community composition in oxic and anoxic sediments. In our study, variations in several environmental factors, such as nutrients (carbon, nitrogen), temperature and salinity, were low at the experimental site^17^. The low background variation facilitated detection of hypoxic-anoxic induced changes in bacterial communities and their interactions with the local benthic ecosystem.

### Abundant bacterial OTUs were most resistant against hypoxic and anoxic disturbance

Abundant OTUs constituted the most stable fraction of the total community during oxygen deficiency, while the moderate fraction reacted most strongly. The response of the rare fraction to prolonged oxygen deficiency was intermediate. These results suggest that the abundant community is adapted to spatially and temporally varying oxygen conditions that typically occur in sediments, whereas moderate and rare communities are more sensitive to hypoxic-anoxic disturbance and reductions in the redox potential of the sediment.

The results of Coveley *et al*.^24^, studying spring sediments, were consistent with our findings in that abundant bacterial communities were most stable against environmental disturbance. The abundance of rare species, in turn, correlated positively with environmental disturbance^20,24^. Abundant microorganisms are likely to be physiologically adapted to environmental conditions such as fluctuating oxygen levels. In contrast, less abundant and rare organisms respond to environmental change such as hypoxic disturbance by changes in population structure (reviewed by^23,49^).

### Abundant communities may possess diverse respiratory strategies during oxygen deficiency

Most of the abundant OTUs were fairly equally present in each treatment and changed slightly only after 48 days of deoxygenation. Thus, oxic (0-day), hypoxic (3-day) and anoxic (7-day treatment) communities considerably overlapped, as noted also by Steenbergh *et al*.^10^. Only the communities of the longest (48 days) deoxygenation treatment differed from the others. The most abundant OTUs belonged to the classes Delta- and Gammaproteobacteria, Actinobacteria and Flavobacteriia, common in coastal sediments worldwide^55^. The composition of abundant OTUs is most likely explained by their adaptation to varying oxygen conditions. Deltaproteobacteria, the most abundant class in our study, was largely represented by taxa of the order Desulfobacteriales that are capable of anaerobic sulfate reduction^56^. However, some taxa in Desulfobacteriales can cope with oxygen^57,58^ or even prefer oxygen as an electron acceptor^59^. Oxygen tolerance could partly explain why DNA from Desulfobacteriales was so abundant also in oxic (0-day treated) sediment.

Gammaproteobacterial species, also abundant in our study, are known to be phenotypically, morphologically and phylogenetically highly diverse^60^. Flavobacteriia and Actinobacteria include species that are able to use various alternative electron acceptors in addition to oxygen^61,62^. Indeed, Actino- and Gammaproteobacteria have been shown to increase in anoxic marine systems^55^, and Flavobacteriia, Gammaproteo- as well as Actinobacteria were abundant in hypoxic and anoxic Baltic Sea sediment^9,63,64^. Thus, these taxa may play an important role in the stability of sediment bacterial communities during oxygen deficiency.

The diversity of respiratory physiology is an example of physiological plasticity, which is essential for the compositional stability of populations^20^. As abundant OTUs were fairly stable, they may also represent metabolically less active cells that are suggested to promote stability against environmental stressors^20^, such as oxygen depletion. However, the effect of extracellular DNA could not be excluded.

### Moderate and rare bacterial fractions changed composition in response to hypoxic and anoxic stress

The moderately abundant fractions of the bacterial communities were clearly shaped by the increasing duration of oxygen deficiency. The distinct tight clustering reflects large compositional changes, i.e. regime shifts from the initial domination of aerobic bacteria to a community of facultative anaerobic bacteria and, ultimately, to obligate anaerobic and fermentative bacteria. Indeed, moderate fractions were comprised of several OTUs that were sensitive or resistant to oxygen depletion. Hypoxia- and anoxia-resistant OTUs can play an important role worldwide, due to the increasing oxygen deficiency in coastal marine ecosystems^3^. Here, sulfate reducing taxa such as the family Desulfobulbaceae and its genus *Desulfophila*^65–67^ proliferated during prolonged oxygen depletion, along with fermenters in the order Clostridiales, such as the genus *Proteocatella*^68,69^. Sulfate reducers use small organic compounds degraded from polymeric compounds by fermenters^56,70^. *Desulfopila* are also capable of fermentation or chemolithoautotrophy^66,67^, which might have contributed to their profusion.

The rare fraction of the bacterial community did not change significantly until after 48 days. Rare members were thus relatively stable, similar to the abundant ones. As opposed to the abundant fraction, the rare fraction could, however, be clearly separated to OTUs sensitive and resistant to oxygen depletion. During prolonged deoxygenation, potential sulfur and sulfate reducers such as the genera *Fusibacter*, *Desulfofrigus* and *Desulfobacter*^65,71,72^ increased along with potential sulfur oxidizers such as the families *Sulfurimonas* and *Arcobacter*^73,74^. Among all rare OTUs, the diversity of rare Deltaproteobacteria, including mainly sulfate reducing taxa, was very high. This may indicate a high potential for sulfate reduction in oxygen-depleted sediments, even in low-salinity brackish systems.

### Temporal variation due to oxygen deficiency

Part of the variation in bacterial community compositions may have been of temporal origin. Nevertheless, the discriminant analysis visualized that the abundance of several moderate and rare OTUs, which were assigned to potential anaerobic taxa, began to increase already after 3 days of deoxygenation. The same taxa became more abundant during 7 days and dominated after 48 days. As 3- and 7-day treatments started at July and 48-day treatment started at June, the systematic increase in these taxa was unlikely due to time but rather due to increasing oxygen depletion and subsequent changes in the redox status in the sediment.

### Moderate and rare bacteria associated with nutrient fluxes across the sediment-water-interface

The OTUs that significantly positively correlated with the efflux of iron, ammonium and silicate from the sediment to the water column almost exclusively originated from the moderate and rare communities. It thus seems that less abundant bacteria were associated either directly or indirectly with nutrient effluxes induced by oxygen deficiency. This supports the recent proposal that the rare biosphere serves as a reservoir of genetic and functional diversity and actively reacts to changes in the ambient environment^23,49^.

The moderate and rare OTUs which positively correlated with nutrient effluxes were frequently the same OTUs that increased during prolonged deoxygenation. For instance, OTUs of Clostridia increased in abundance along with an increasing ammonium efflux. A mechanism behind the positive correlation of Clostridia with ammonium flux could be its capacity to ferment organic compounds to ammonium^75^. OTUs in the phylum Bacteroidetes also correlated positively with ammonium. Although not well described, Bacteroidetes may also harbour similar proteolytic activities in marine sediments as has been shown for the human gut^76^, which could explain their correlation with ammonium. Members of Bacteroidetes have been found to contribute to dissolution of biogenic silica and are often associated with diatoms^77–79^, which may explain their correlation with silicate. The silicate efflux, however, may have been accelerated due to degradation of diatoms, as they most probably died without oxygen and light during deoxygenation^17^.

OTUs in the class Clostridia, the order *Desulfuromonadales* and its genus *Geobacter* correlated positively with the iron efflux. Several iron-reducing species of Clostridia, *Desulfuromonadales* and *Geobacter* are known and have been found in marine sediments^80–82^. The efflux of iron from our sediments may have been accelerated by these bacteria.

### Variation in alpha and beta diversity marked the threshold response to hypoxic and anoxic stress

Probably due to higher resolution of sequencing, the Shannon H’ diversity of 16S rRNA gene sequences revealed more variation across treatments than Shannon H’ of T-RFLP data. The diversity of 16S rRNA gene sequences slightly decreased after three days and increased after seven days of deoxygenation. Most probably, the decrease in the diversity after three days was due to the extinction of aerobes that could not tolerate the oxic-anoxic transition. After seven days, anoxic conditions enriched anaerobes and facultative anaerobes that can use alternative electron acceptors instead of oxygen. The slight recovery of OTU diversity after seven days indicates that a part of the community, most likely facultative anaerobes, could be resilient against shorter periods of hypoxia or anoxia. Ultimately, after a longer period of anoxia, the diversity of both T-RFs and OTUs radically diminished indicating that only anaerobes adapted to low redox potential could live. Beta diversity, describing the variance in the community, responded to oxygen deficiency in an opposite manner. Variance increased significantly after 3 and 48 days of oxygen depletion but decreased after 7 days, probably caused by the observed community switch described above. Long-term exposure caused most heterogeneity in communities, suggesting that prolonged disturbance made communities vulnerable. To conclude, the community went through a regime shift from high- to low-diversity composition, along with increased instability. The results indicate that the threshold for disturbance, in form of oxygen deficiency, was exceeded.

### Relationships between benthic macrofaunal and bacterial diversity during hypoxic and anoxic stress

The alpha diversity of the bacterial community and the benthic macrofaunal diversity correlated strongly, and positively. Interestingly, benthic faunal diversity did not correlate with the diversity of abundant and moderate OTUs but rather with the rare ones. The rare OTUs represented several taxa, sensitive to oxygen deficiency, which explains the increasing effect of faunal diversity on bacterial diversity. The correlation between the benthic fauna and OTUs that are sensitive to oxygen deficiency is consistent with previous findings^83,84^. According to these, microbial communities which were associated with burrows created by bioturbating animals were similar to microbial communities in oxic surface sediments.

Due to the overlapping effect of the deoxygenation treatments, the direct effect of benthic faunal diversity on bacterial diversity was 3%, similar to recently observed levels^85^. Although the effect of faunal diversity on bacterial diversity was small, it was very important. During the first seven days of deoxygenation, the bacterial alpha diversity remained stable likely through bioturbation, which decreased but did not totally cease owing to the relatively hypoxia-resistant species such as *Macoma balthica* and *Mya arenaria*^17^. We assume that the sharp drop in bacterial alpha diversity by day 48 was triggered by the complete lack of benthic fauna and their biological functions, including bioturbation and bioirrigation, especially by large macrofauna^17,86^. Bacterial diversity dropped only when the benthic fauna was eliminated. Thus, bioturbation may have maintained at least a fraction of the bacteria sensitive to a very low redox potential.

### Ecological consequences of hypoxia-induced changes in bacterial communities

Prolonged deoxygenation increased the number of hypoxia and anoxia resistant bacterial taxa. From the perspective of shallow coastal ecosystems, hypoxia- and anoxia-resistant bacteria are principally deleterious as they harm eukaryotes and promote the release of nutrients from the sediment to the water column. For instance, H_2_S, produced by sulfate reducers, damages most eukaryotic organisms such as benthic macro- and meiofauna, which ultimately leads to a cessation of bioturbation^2,15^. In the absence of bioturbation, oxygen transport into the sediment is prevented, inhibiting oxygen-dependent nitrification and subsequently nitrate-dependent denitrification, which cause ammonium to accumulate^87^.

Our results also showed that the changes in bacterial communities were associated with effluxes of iron and silicate, which increased with prolonged periods of oxygen deficiency. The hypoxia-induced and likely microbially mediated efflux of ammonium, iron and silica (recently shown by^88^), can accelerate eutrophication due to the bioavailability of these nutrients for bacteria and algae. Eutrophication, in turn, increases hypoxia on the seafloor, which enhances e.g. flux of redox-sensitive phosphorus and a further proliferation e.g. of nitrogen-fixing cyanobacteria^89^. Microbial reduction of iron oxyhydroxides may also directly release phosphorus that would otherwise be immobilized in the sediment when bound to iron^90^. It is important to note that benthic bioturbation may maintain the diversity of hypoxia- and anoxia-sensitive bacteria that have oxygen-dependent functions and can reduce nutrients in aquatic systems. For example, coupled nitrification-denitrification, which enables removal of nitrogen from water bodies, is facilitated by bioturbating animals^91^. Another example is the oxidation of ferrous iron that enables immobilization of phosphorus from the sediment.

## Conclusions

The sensitivity of moderate and rare OTUs to oxygen depletion and subsequent changes in the sediment redox potential resulted in a regime shift from aerobic to obligate anaerobic taxa by 48 days of deoxygenation. The regime shift potentially resulted in functional changes in communities that may cause harmful effects on marine ecosystem, such as higher nutrient effluxes.

High bacterial diversity was a key factor for maintaining the resistance of the communities under hypoxic stress until it dropped after 48 days of oxygen deficiency. That is, the threshold for community stability was exceeded. If hypoxia occurs for shorter periods, or there is even a slight improvement in bottom-water oxygen concentration, bacterial communities may maintain their stability with the help of benthic fauna and recover fast if the bottom water returns normoxic. The current and previous studies^17,92^ thus propose that the benthic ecosystem of coastal sandy sediments remains resilient during short periods of hypoxia, while longer periods of oxygen deficiency are highly disruptive. For the functioning of coastal ecosystems such as the Baltic Sea, it is therefore crucial to prevent episodic hypoxia to progress to persistent anoxia. Shortening of hypoxic periods should be among the primary goals in the protection of coastal water bodies. In shallow areas with a stratified water column, hypoxia often occurs from two to four months annually^93^, and a reduction in external loading of nutrients and organic matter is needed to remediate this.

## Supplementary information

Supplementary information
