# Supplementary material for: Increasing oxygen deficiency changes rare and moderately abundant bacterial communities in coastal soft sediments
Source: Sci Rep. 2019 Nov 8;9:16341. doi: 10.1038/s41598-019-51432-1 (PMC6841974; doi:10.1038/s41598-019-51432-1)
Supplement: Supplementary file 1 — Supplementary information [file 41598_2019_51432_MOESM1_ESM.docx]

**SUPPLEMENTARY INFORMATION**

**The manuscript title:**

Increasing oxygen deficiency changes rare and moderately abundant bacterial communities in coastal soft sediments

**Authors:**

Hanna Sinkko, Iina Hepolehto, Christina Lyra, Johanna M. Rinta-Kanto, Anna Villnäs, Joanna Norkko, Alf Norkko and Sari Timonen

This file includes eigth supplementary figures (pages 2-5) and a supplementary table (Supplementary Table 1, page 7), which are referred to in the main manuscript.

**Supplementary Figure 1** Operational taxonomic units (OTUs, n=19 777) of 16S rRNA gene sequences, normalized by variance stabilizing transformation. Categories of abundant, moderate and rare OTUs, as well as noise were formed based on statistical examination (refer to Figure 1).


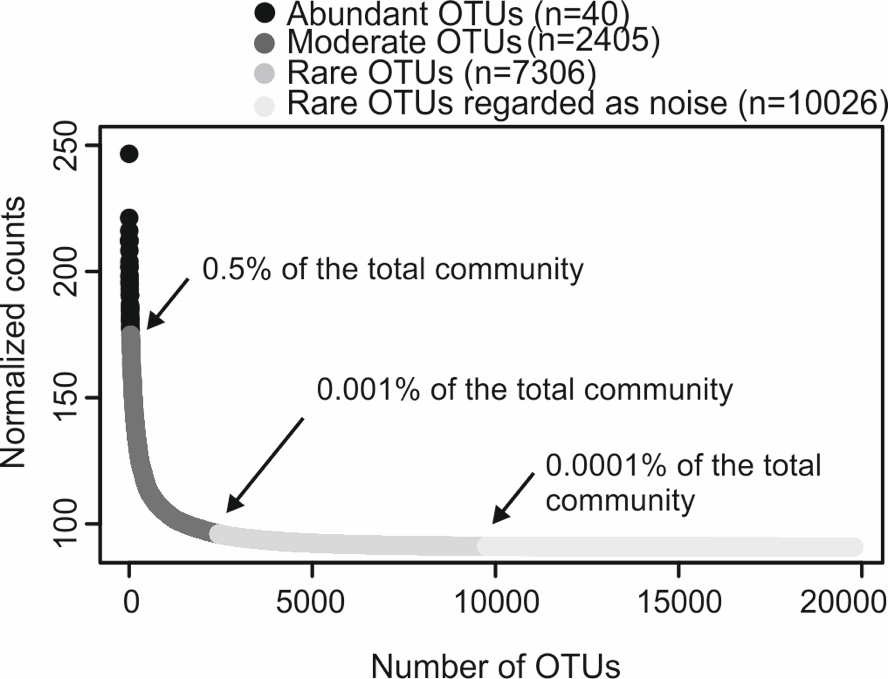


**Supplementary Figure 2** Quantities of bacterial 16S rRNA gene copies in sandy sediments during deoxygenation of the Baltic Sea seafloor. Eight samples were analyzed for each duration (day, d) of deoxygenation.


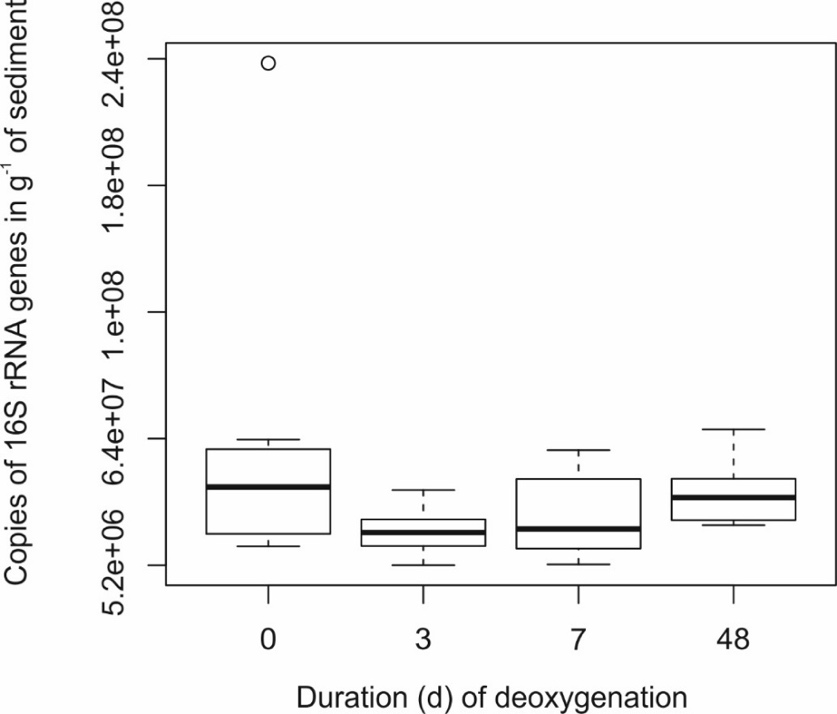


**Supplementary Figure 3** Differences between bacterial community compositions of sandy sediments that were deoxygenated *in situ*. The compositions (n = 32; including duplicates per treatment plot to show within - treatment plot variation) were based on terminal restriction fragments (T-RFs) of bacterial 16S rRNA genes created by the restriction endonuclease (A) HaeIII and (B) RsaI. Ordinations for T-RFs were constructed using discriminant axes scores produced by nonparametric linear discriminant analysis, which was based on Bray-Curtis dissimilarity between samples. Ticks on the top of symbols depict samples selected for sequencing of the 16S rRNA genes. Abbreviations: d = day.


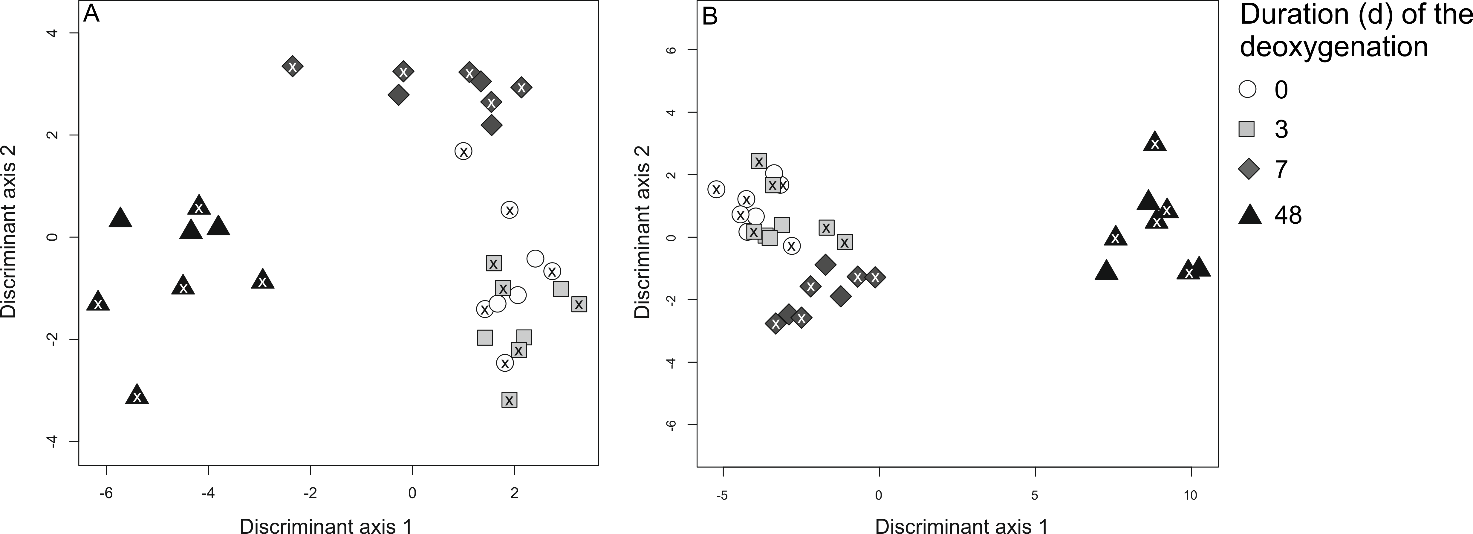


**Supplementary Figure 4** The heterogeneity of variances in bacterial community compositions during deoxygenation of the seafloor. Terminal restriction fragments of bacterial 16S rRNA genes of sediments created by the restriction endonuclease (A) HaeIII and (B) RsaI (n = 32). The variances for different deoxygenation treatments were calculated using the Bray-Curtis based nonparametric test for homogeneity of multivariate group dispersions. Values in squared boxes represent *p*-values for pairwise comparisons between deoxygenation treatments, produced by 9999 permutations of model residuals. Abbreviations: d = day.


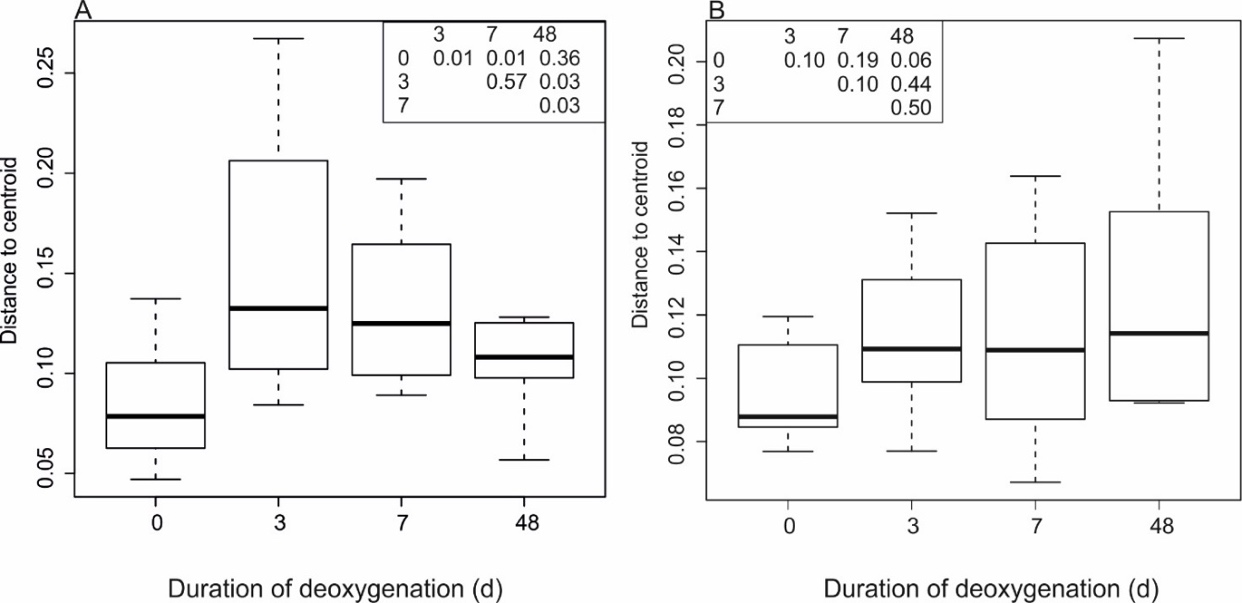


**Supplementary Figure 5** Differences between the bacterial 16S rRNA gene compositions during deoxygenation of the seafloor. Ordination plots for (A) abundant OTUs and (B) total OTUs, excluding noisy OTUs, were constructed using scores of discriminant axes 1 and 2 that were produced by nonparametric linear discriminant analysis. The discriminant analyses used (A) 4 and (B) 2 principal coordinates, based on the Bray-Curtis dissimilarity index, which explained 83% and 21% of total variation, respectively. (A) 46% (*p* = 0.14) and (B) 70% (*p*=0.003) of the community compositions (n = 20) were classified based on the duration of deoxygenation. Abbreviations: d = day. Ticks on the top of symbols mark within – treatment plot replicates showing their variation

**Supplementary Figure 6** Percentages of taxonomic classes represented by differentially abundant fractions of bacterial communities. (**a**) Abundant, (**b**) moderate and (**c**) rare OTUs in all treatments were assigned to a class. The number above each bar represents the number of different OTUs in the corresponding class. Moderate and rare classes represented only by one or two OTUs are not shown as their percentage was very low (<0.0001%). Among the abundant, moderate and rare communities, classes that could not be assigned to any known taxa accounted for 6.0%, 13.3%, and 1.4%, respectively.


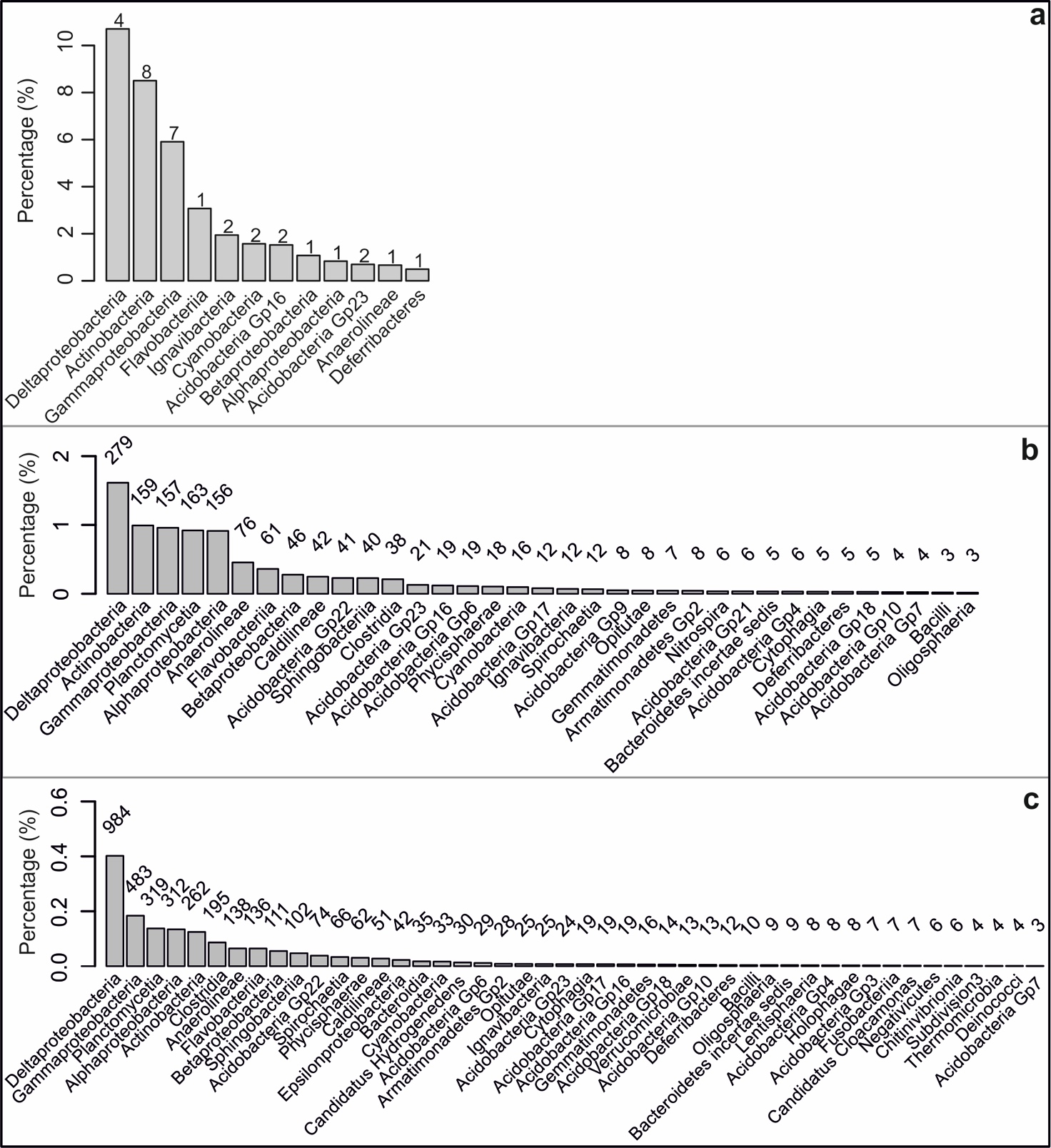


**Supplementary Figure 7** Shannon H’ diversity indices of differentially abundant fractions of bacterial communities. (A) Abundant, (B) moderate and (C) rare fractions of the total community. Abbreviations: d = day.


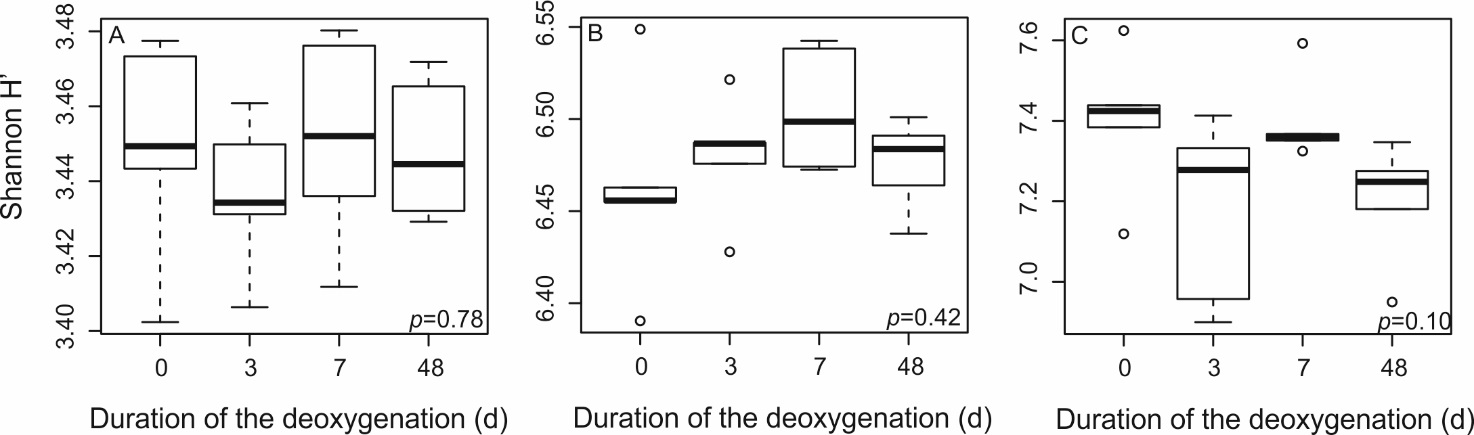


**Supplementary Figure 8** Partitioning of variance in the Shannon H’ index of bacterial 16S rRNA gene sequence data into proportions affected by diversity of benthic macrofauna and duration of hypoxia as well as their interactions.


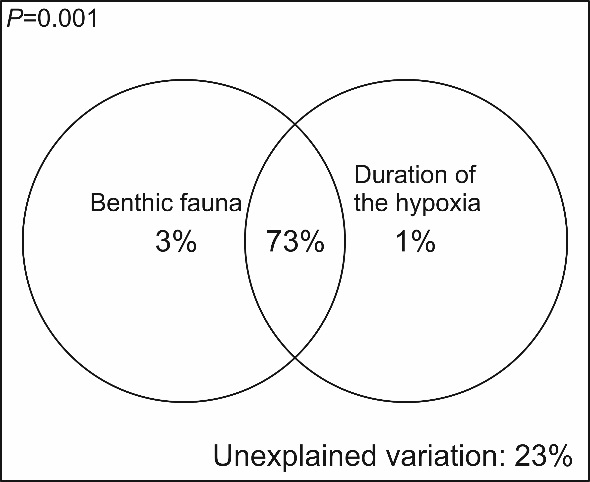


**Supplementary Table 1** Global analysis of variance and pairwise comparisons of bacterial communities between different durations of deoxygenation (treatments). Bacterial community compositions were based on terminal restriction fragments (T-RFs) of 16S rRNA genes created by the restriction endonucleases HaeIII and RsaI. Mean abundances of each OTUs were calculated from within - treatment plot duplicates before the analysis. The variance analysis used Bray-Curtis dissimilarity between samples and 9999 permutations of raw data to calculate *p*-values. *P* values were adjusted using the false discovery rate (FDR) method.

| **T-RFs created by HaeIII** | | | | | |  |
| --- | --- | --- | --- | --- | --- | --- |
| **Treatments** | **SS** | **MS** | **F. Model** | **R^2^** | **Pr (>F)** | ***P* adjusted** |
| 0 vs. 3d | 0.02 | 0.02 | 1.41 | 0.19 | 0.1715 | 0.1715 |
| 0 vs. 7d | 0.04 | 0.04 | 2.75 | 0.31 | 0.0271 | 0.0420 |
| 0 vs. 48d | 0.25 | 0.25 | 39.57 | 0.87 | 0.0280 | 0.0420 |
| 3 vs. 7 d | 0.05 | 0.05 | 2.05 | 0.25 | 0.0871 | 0.1016 |
| 3 vs. 48 d | 0.22 | 0.22 | 15.11 | 0.72 | 0.0292 | 0.0420 |
| 7 vs. 48 d | 0.14 | 0.14 | 10.78 | 0.64 | 0.0300 | 0.0420 |
| Global test | 0.36 | 0.12 | 8.17 | 0.67 | 0.0001 |  |
| **T-RFs created by RsaI** | | | | | |  |
| **Treatments** | **SS** | **MS** | **F. Model** | **R^2^** | **Pr (>F)** | ***P* adjusted** |
| 0 d vs. 3d | 0.01 | 0.01 | 1.68 | 0.22 | 0.0818 | 0.0818 |
| 0 d vs. 7d | 0.03 | 0.03 | 2.83 | 0.32 | 0.0301 | 0.0359 |
| 0 d vs. 48d | 0.19 | 0.19 | 23.21 | 0.79 | 0.0308 | 0.0359 |
| 3 d vs. 7 d | 0.02 | 0.02 | 1.96 | 0.25 | 0.0253 | 0.0359 |
| 3 d vs. 48 d | 0.14 | 0.14 | 17.70 | 0.75 | 0.0291 | 0.0359 |
| 7 d vs. 48 d | 0.14 | 0.14 | 12.56 | 0.68 | 0.0284 | 0.0359 |
| Global test | 0.27 | 0.09 | 9.64 | 0.71 | 0.0001 |  |
